# Evaluating the principles of radiation protection in diagnostic radiologic examinations: collimation, exposure factors and use of protective equipment for the patients and their companions

**DOI:** 10.1002/jmrs.384

**Published:** 2020-03-09

**Authors:** Zahra Farzanegan, Marziyeh Tahmasbi, Mohsen Cheki, Fatameh Yousefvand, Mohammad Rajabi

**Affiliations:** ^1^ Faculty of Medicine Department of Medical Physics Ahvaz Jundishapur University of Medical Sciences Ahvaz Iran; ^2^ Faculty of Paramedicine Department of Radiologic Technology Ahvaz Jundishapur University of Medical Sciences Ahvaz Iran

**Keywords:** Collimation, exposure factors, patient companion, radiation field, radiation protection

## Abstract

**Introduction:**

Producing appropriate diagnostic images along with patient radiation protection is the goal of radiography. Due to the advancements of radiography, concerns about observing the principles of radiation protection exist. Therefore, this study aimed to evaluate the observance of the principles of radiation protection in radiographic examinations with emphasis on field size collimation, suitability of exposure factors and the use of protective equipment for the patients and their companions.

**Methods:**

Using a cross‐sectional study design, two radiography students on their final year of study observed 100 radiographic examinations from the imaging departments of five educational hospitals. The SPSS version 24 software was used to analyse the results.

**Results:**

The radiation field collimation was obtained in 46% of the studied radiographs. Patients had companions present during the examination in 26% of the studies; however, protective equipment was only used for 4% of the patients’ companions, and no protective equipment was applied for patients. The observance rate of the various principles of radiation protection including field size restriction, the use of protective equipment for the patients and their companions, and suitability of the selected exposure factors was on average 44.6%.

**Conclusion:**

The observance rate of the principles of radiation protection was insufficient in the studied educational hospitals, specifically in field size collimation and the use of protective equipment for the patients and their companions. Therefore, emphasis on the strict implementation of the radiation protection guidelines and continuous training of radiographers are required.

## Graphical abstract

This study aimed to evaluate the observance of the principles of radiation protection in radiographic examinations. Field size collimation, suitability of exposure factors and the use of protective equipment for the patients and their companions were done on average 44.6% in the reviewed examinations.

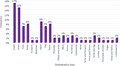

## Introduction

With the advancement of medical imaging devices, diagnostic imaging techniques to detect different diseases are increasing rapidly.^1^, ^2^ The goal of radiography is to produce appropriate diagnostic images to establish the appropriate diagnosis while simultaneously protecting patients against radiation. The increasing incidence of X‐ray examinations raises concerns about the full observance of the principles of radiation protection by radiographers^3^ because exposure to an excessive amount of ionising radiation can have negative effects on the hematopoietic system, the central nervous system and ultimately the overall system of the human body, and the intensity of these effects depends on the age of the patient, the exposed anatomical region and the received doses.^4^

During radiologic examinations, several factors are controlled by the radiographers, which can maximise the diagnostic value of the image and minimise patient exposure.^5^ Various methods such as the use of lead shields, lead glasses and lead aprons, short exposure times, proper source to image receptor distance and use of three‐phase generators and intensifying screen are recommended to reduce the patient's radiation dose specifically for the radiation‐sensitive organs such as the thyroid, eye lens and gonads.^4^ Moreover, beam energy, filtration, field size and tissue thickness are factors that affect patient's radiation doses in several imaging methods.^6^

Applying the principles of radiation protection can prevent the deterministic effects of ionising radiation and decrease the related stochastic effects. Although several protective measures are considered significantly simple, radiographers’ proper observance of these measures eliminates most of the unwanted and unnecessary radiation hazards.^6^

To the best of our knowledge, localisation of radiation field is one of the principles required to reduce patient's radiation doses.^7^, ^8^ During radiographic examination, the area of the patient's body exposed to X‐rays should be limited to the target area which is under consideration of medical examination.^6^, ^8^, ^9^ As the tissues exposed to primary radiation frequently receive a significantly higher amount of radiation doses compared to tissues outside the radiation field, the amount of unnecessary exposure can be significantly decreased by beam collimation. Accordingly, radiation doses in the skin and internal tissues will be decreased.^6^, ^10^

Moreover, the use of lead aprons and protective equipment for the patients and their companions, in cases where the presence of companions during radiographic examination is necessary, is significantly important.^4^ Therefore, this study aimed to evaluate the observance of the principles of radiation protection in imaging centres of educational hospitals with emphasis on field size restriction, suitability of the selected exposure factors and the use of protective equipment for patients and their companions during radiologic examinations.

## Methods

This was a cross‐sectional descriptive–analytic epidemiologic study which was approved by Ahvaz Jundishapur University of Medical Sciences ethics committee and the institutional review board based on the proposed research plan. The managers of the educational hospitals and the imaging departments provided informed consent for inclusion in the study. Two of the researchers on their apprenticeships in the educational hospital included in this study were also the observers; hence, their presence and observation of the imaging process was usual, and the radiographers who were also the students’ educators at the imaging centres were aware that they were observed. Also, patients provided informed consent for inclusion in the study.

General imaging departments of five educational hospitals were included in the study, and 100 conventional radiologic examinations were evaluated. The sample size was selected based on the previous study.^11^ Data were collected by fourth year radiography students who were at their final year of undergraduate degree and were passing their practical courses at the training hospitals (refer to the Supporting Information for the data collection sheet). They were instructed to go to the radiography centres of the five educational hospitals at one work shift (morning work shift) daily for approximately 1 month. Different patients who underwent radiographic examinations, including adults and children, were selected based on convenience sampling, and the prepared checklists were completed by direct observation of radiography for patients who provided informed consent.

The SPSS version 24 software was applied to analyse the results. Score of 1 for Yes and zero for No was considered in the yes/no questions. The Kolmogorov–Smirnov test was used to analyse the normality of data. Due to non‐normality of data, the Mann–Whitney U‐test was used. The Kruskal–Wallis test was used to examine the association among the investigated variables (the association between the studied patients’ demographic characteristics and the following parameters: type of radiography, referral cause, suitability of the adjusted exposure factors and field size, and the presence of patients’ companions). *P*‐value < 0.05 was considered statistically significant.

## Results

The reviewed radiographic examinations were obtained by eight radiographers in five educational hospitals, including five men and three women with a mean age of 42.6 ± 3.2 years. All radiographers were official employees of the hospitals with a mean experience duration of 16.4 ± 2.9 years. One of the radiographers had Master of Science degree in anatomy, and the remaining radiographers had Bachelor of Science degrees in radiography.

Based on the results, the mean age of the studied patients was 32.3 ± 17.1 years, and among the reviewed radiographs, 64% were for men and 36% for women.

An average of 33.3% of the observed radiographs was obtained by the analogue X‐ray units, and similar percentages of the reviewed images were obtained by digital radiology (33.3%) and computed radiology (33.3%).

In 64% of the studied radiographs, the radiation exposure factors were set manually, and automatic exposure control was performed in 36% of cases.

**Figure 1:**
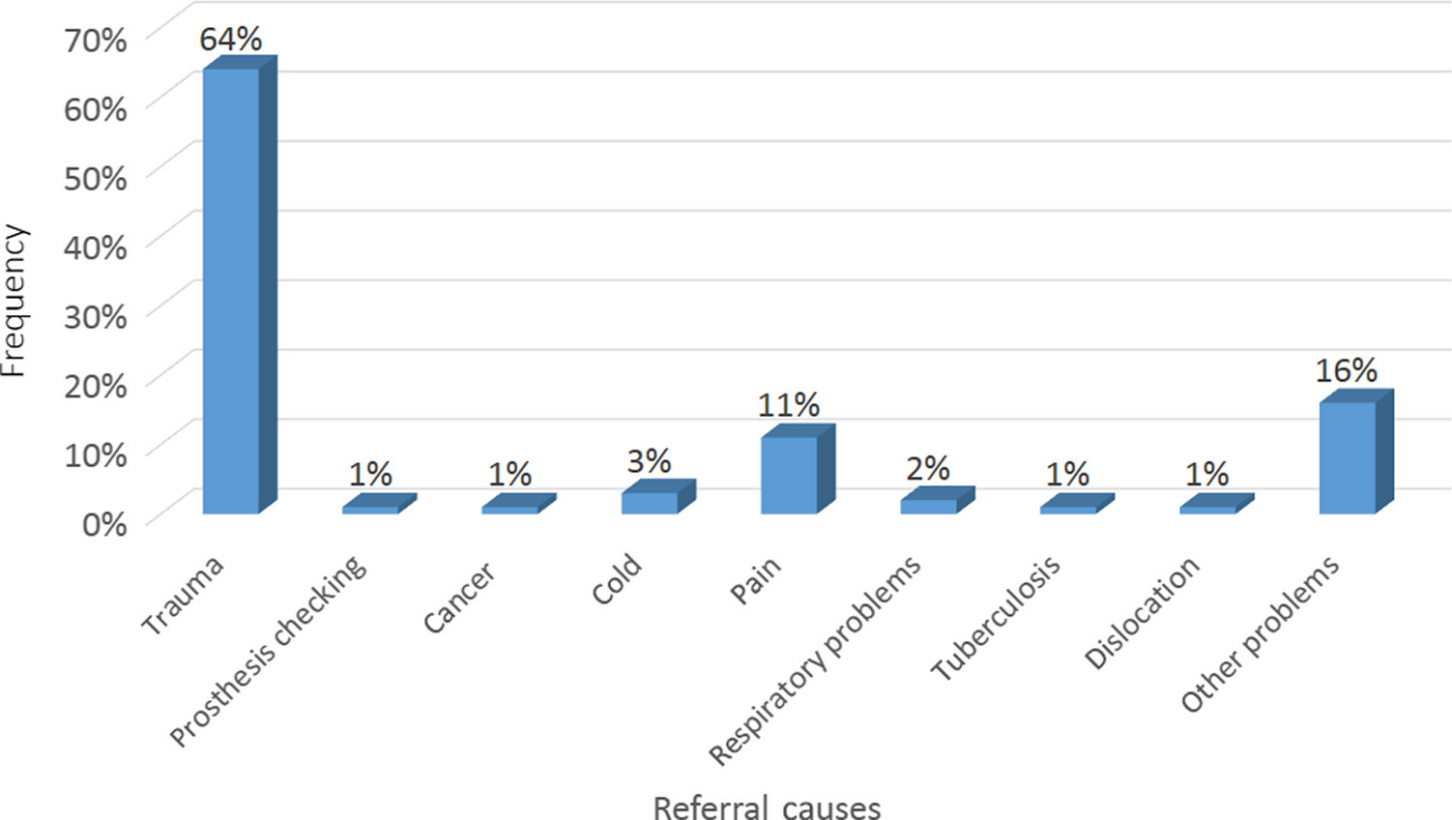
The causes for referring the patients to the imaging centre in the studied radiographs.

**Figure 2:**
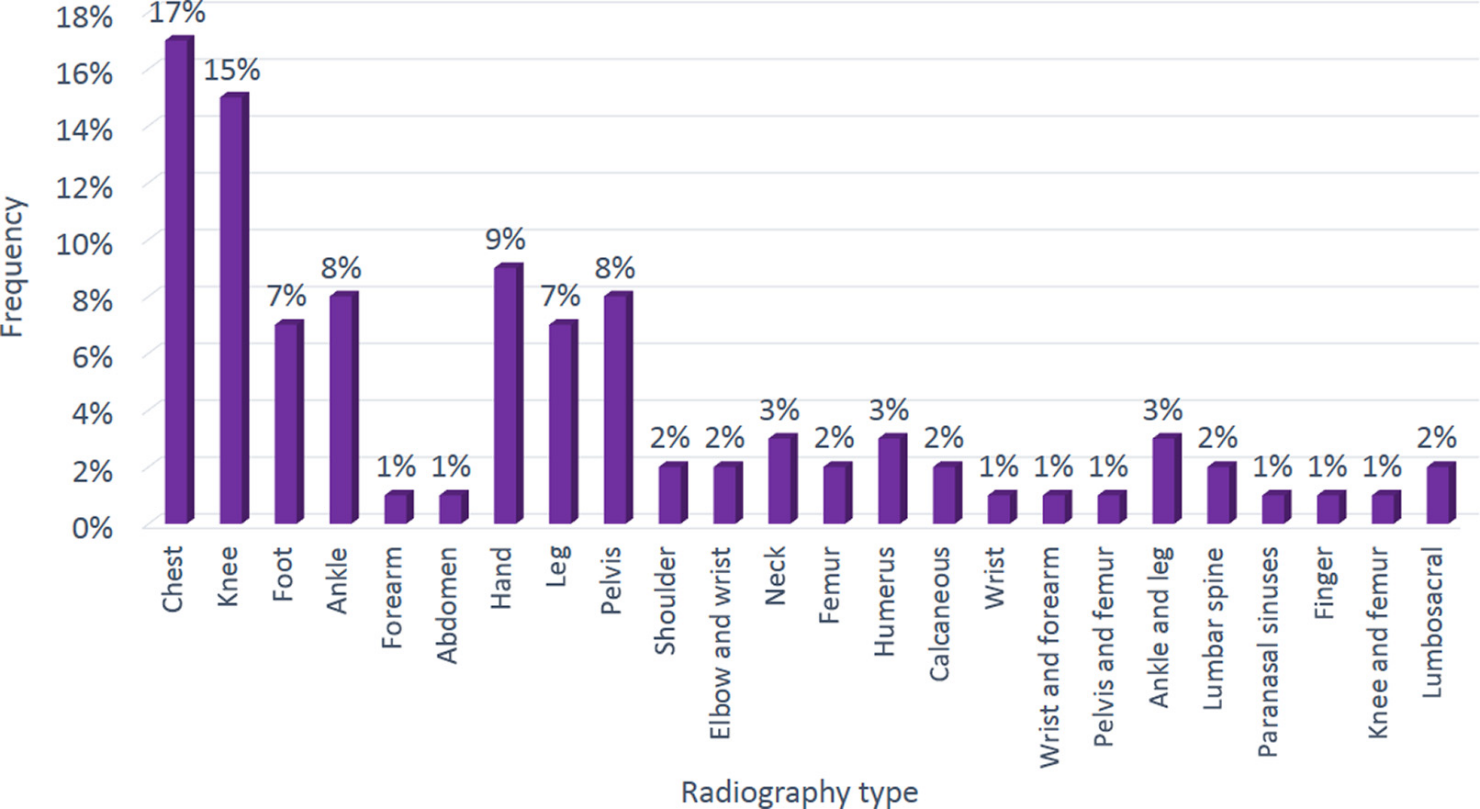
The percentages of the different studied radiographs.

Reasons for referring the patients to the radiology centres are presented in Figure 1, and different types of studied radiographs are illustrated in Figure 2.

The average, maximum and minimum radiation field sizes used for the various studied radiographs are shown in Table 1. The standard field sizes for different radiographic examinations are also indicated in this table according to *Merrill's Atlas of Radiographic Positioning & Procedures* which is used as a guideline for our radiology departments.^12^

**Table 1:** The average, maximum, minimum and the standard field sizes for studied radiographies according to Merrill's Atlas.^12^

Studied organ	Average field size (cm^2^)	Maximum applied field size (cm^2^)	Minimum applied field size (cm^2^)	Standard field size (cm^2^)
Chest	35 × 43	25 × 30	60 × 60	35 × 40
Knee	24 × 40	15 × 60	20 × 50	15 × 28
Foot	24 × 30	15 × 20	30 × 40	15 × 28
Ankle	24 × 35	20 × 25	30 × 40	10 × 23
Forearm	40 × 50	40 × 50	40 × 50	13 × 38
Abdomen	60 × 60	60 × 60	60 × 60	35 × 43
Hand	24 × 30	15 × 20	40 × 50	18 × 20
Leg	40 × 50	20 × 40	80 × 30	15 × 43
Pelvis	35 × 45	15 × 40	35 × 45	35 × 43
Shoulder	45 × 45	40 × 40	50 × 50	28 × 23
Elbow & wrist	45 × 55	40 × 50	50 × 60	13 × 38
Neck	20 × 25	15 × 20	25 × 30	13 × 25
Femur	40 × 50	35 × 45	40 × 50	20 × 43
Humerus	40 × 35	45 × 25	30 × 40	18 × 43
Calcaneus	20 × 40	20 × 40	20 × 40	10 × 15
Wrist	25 × 30	25 × 30	25 × 30	20 × 20
Wrist and forearm	20 × 25	20 × 25	20 × 25	13 × 38
Pelvis and femur	35 × 45	35 × 45	35 × 45	35 × 43
Ankle and leg	35 × 35	15 × 40	43 × 17	15 × 43
Lumbar spine	20 × 40	20 × 40	20 × 40	23 × 35
Paranasal sinuses	25 × 30	25 × 30	25 × 30	15 × 15
Finger	25 × 30	25 × 30	25 × 30	5 × 15
Knee and femur	25 × 50	25 × 50	25 × 50	20 × 43
Lumbosacral	35 × 55	20 × 40	50 × 70	25 × 20

The standard field sizes for the studied radiographs is obtained from Merrill's Atlas 12.

Table 2 presents the mean, maximum and minimum values of mAs and peak kilovoltage (KVp) used in the various studied radiographs. The standard values of these quantities according to *Merrill's Atlas of Radiographic Positioning & Procedures* 12 are also indicated in this table. Moreover, this table shows the KVp decrement percentages and the related maximum mAs, which is acceptable for various studied radiologic examinations according to the following principle: radiographer can double the mAs by 15%, decreasing the KVp 13. Accordingly, a number of cases in which the adjusted mAs and KVp were higher than the permitted values were calculated and are also shown in this table. Furthermore, this table indicates the percentages for suitability of adjusting the KVp and mAs in the studied radiologic examinations.

**Table 2:** The mean, maximum, minimum and the standard values of mAs and kVp, maximum accepted mAs with respect to KVp decrement (ΔKVp%), number of cases with mAs and KVp higher than the accepted values and the percentages for suitability of adjusted mAs and KVp applied for various studied radiographs.

Studied organ	Total studied cases	Average ± SD of the implemented KVp	Maximum applied KVp	Minimum applied KVp	Standard KVP	Cases with KVp higher than the standard	Suitability of adjusted KVp (%) For each examination	ΔKVp (%)	Maximum accepted mAs	Average ± SD of the implemented mAs	Maximum applied mAs	Minimum applied mAs	Standard mAs	Cases with mAs higher than the accepted values	Suitability of adjusted mAs (%) For each examination
Chest	17	69 ± 5	80	60	120	0	100	43	18	28 ± 9	40	7	3	14	18
Knee	15	54 ± 7	70	45	70	0	100	23	15	7 ± 2	12	4	5	0	100
Foot	7	49 ± 5	55	44	70	0	100	30	10	4 ± 2	6	2	3	0	100
Ankle	8	50 ± 9	68	45	70	0	100	30	13	5 ± 1	6	3	3	0	100
Forearm	1	50 ± 0	50	50	70	0	100	30	9	4 ± 0	4	4	2	0	100
Abdomen	1	70 ± 0	70	70	85	0	100	18	60	60 ± 0	60	60	25	0	100
Hand	9	50 ± 11	74	40	66	1	89	24	5	4 ± 3	10	1	2	2	78
Leg	7	55 ± 8	70	50	70	0	100	21	5	6 ± 5	16	4	4	2	71
Pelvic	8	68 ± 3	70	62	85	0	100	20	63	42 ± 15	60	25	25	0	100
Shoulder	2	55 ± 7	60	50	85	0	100	35	46	8 ± 6	13	4	10	0	100
Elbow and wrist	2	44 ± 0	44	44	70	0	100	37	11	2 ± 0	2	2	2	0	100
Neck	3	62 ± 7	70	56	85	0	100	27	23	15 ± 15	32	4	6	1	67
Femur	2	69 ± 1	70	69	88	0	100	21	21	32 ± 0	32	32	7	2	0
Humerus	3	61 ± 4	66	59	70	0	100	13	7	25 ± 17	45	15	4	3	0
Calcaneus	2	50 ± 0	50	50	70	0	100	29	12	3 ± 3	5	1	3	0	100
Wrist	1	49 ± 0	49	49	66	0	100	26	7	5 ± 0	5	5	2	0	100
Wrist and forearm	1	52 ± 0	52	52	70	0	100	26	8	5 ± 0	5	5	2.2	0	100
Pelvic and femur	1	66 ± 0	66	66	85	0	100	23	75	20 ± 0	20	20	25	0	10
Ankle and leg	3	54 ± 2	57	52	70	0	100	23	11	7 ± 2	10	5	3.6	0	100
Lumbar spine	2	72 ± 2	73	70	90	0	100	21	60	66 ± 43	96	35	20	1	50
Paranasal sinus	1	68 ± 0	68	68	85	0	100	9	17	32 ± 0	32	32	16	1	0
Finger	1	40 ± 0	40	40	63	0	100	37	8	2 ± 0	2	2	1.6	0	100
Knee and femur	1	56 ± 0	56	56	87	0	100	36	36	13 ± 0	12	12	7.1	0	100
Lumbosacral	2	70 ± 0	70	70	90	0	100	22	84	25 ± 22	40	9	28	0	100
Total percentage (%)	‐	‐	‐	‐	‐	1%	99%	‐	‐	‐	‐	‐	‐	26%	74%

The related maximum mAs accepted for various studied radiology examination is calculated according to the law which let the radiographers to double the mAs with 15% decreasing the KVp.^13^

The standard values of the KVp and mAs for each radiography are obtained from the Merrill's Atlas.^12^

The presence of various protective equipment in the studied radiology centres is shown in Table 3. The percentages of using the protective equipment for the patients and their companions and the percentages of selecting suitable exposure factors and field size restriction in the observed radiographic examinations are illustrated in Table 4.

**Table 3:** Existence of the different protective shields in the radiology centres of the studied educational hospitals.

Hospital	Thyroid	Gonad	Lead gloves	Lead apron	Lead glasses	Lead paravan
A	✓	✓	✓	✓	✓	✓
B	✓	✓	✓	✓	✓	✓
C	✓	‐	‐	✓	‐	‐
D	✓	✓	‐	✓	✓	✓
E	✓	✓	‐	✓	‐	✓

**Table 4:** Percentages for different protective measures (including: proper field size restriction, suitable exposure factors selecting, patient and the companion shielding).

Patient shielding^a^	Companion shielding^a^	The selected mAs suitability^b^	The selected KVp suitability^b^	Proper field size restriction^b^
0%	4%	74%	99%	46%

a According to observation

b According to the Merrill's reference.^12^

The association between age ranges and the gender of patients based on the type of the requested radiography, mAs and KVp values, suitability of selected mAs, KVp, the correct field size collimation and the presence of companions in the examination room during the radiography are shown in Table 5. The association between the type of the requested radiography and the parameters mentioned above is also shown in this table.

**Table 5:** P‐values for the association of patients’ demographic characteristics and the radiography type with the studied parameters (mAs and KVp values, suitability of mAs, KVp, and field size, presence of companion during the radiography).

	Radiography type	mAs value	KVp value	mAs suitability	KVp suitability	Suitability of Field size	Presence of companion
Age range	0.01*	0.19	0.60	0.30	0.35	0.32	0.03*
Gender	0.69	0.39	0.04*	0.86	0.18	0.51	0.76
Radiography type	‐	0.00*	0.00*	0.00*	0.99	0.03*	0.50

* shows the significant association

## Discussion

An observational cross‐sectional descriptive–analytic epidemiologic study was conducted to evaluate the principles of radiation protection performance of radiographers. A total of 100 conventional radiologic examinations performed by eight radiographers in five educational hospitals were selected based on convenience sampling and reviewed using a checklist by two radiography students on their apprenticeships in the hospitals included in the study.

Results showed that trauma (64%) was the most common reason for referring patients to the radiology centres, while tuberculosis, cancer follow‐up, bone dislocation and prosthetic examinations with an average of 1% were the least common reasons. Chest (17%) and knee (15%) X‐rays had the highest percentages of the requested radiographies, while the finger, paranasal sinus, abdomen and forearm X‐rays (with an average of 1%) had the lowest frequency.

The association between patient's age and the type of requested radiography was significant (*P*‐value = 0.03). Moreover, most of the patients who underwent radiography were in the age range of 21–30 years, and the abdomen, upper and lower limbs radiographies were highly requested for them. Furthermore, the chest radiography was highly requested for the age range of 31–40 years, while radiography in lumbosacral region was highly requested for age range of 41–50 years and spine imaging was more requested for patients in age range of 11–20 years.

Findings revealed that the average of the applied mAs was greater than acceptable value for 26% of the reviewed examinations. Moreover, the highest differences between the mean adjusted mAs and maximum permitted value for that certain radiographic examination with respect to the selected KVp were observed in humerus, paranasal sinus, femur and chest imaging. The maximum difference percentage was for humerus. These findings suggest that radiographers were more concerned about the image quality than the patient's doses.

Furthermore, the association between the type of the requested radiography and the value of adjusted mAs was significant (*P*‐value = 0.0), which seems to be evident. For example, the highest values of mAs were used for lumbar spine and abdominal radiographies with an average of 66 and 60 mAs, respectively. Furthermore, the requested radiography type had a significant association with suitability of adjusted mAs (*P*‐value = 0.0); hence, the least percentage of mAs appropriateness was observed in chest radiography (18%).

Based on our results, the average values of the selected kilo‐voltages were more appropriate for the studied radiographs (99% suitability of KVp setting). As expected, KVp had a significant association with the radiography type (*P*‐value = 0.0); hence, the highest levels of KVp are used for lumbar spine, lumbosacral and chest imaging, respectively, because these organs are relatively thick.

Furthermore, our results presented a statistically significant association between the patient's gender and the selected KVp (*P*‐value = 0.04). Hence, higher values of KVp were used for men.

Moreover, according to the results, field size collimation was observed only in 46% of the reviewed radiologic examinations compared *to Merrill's Atlas*.^12^ This finding is consistent with the results of Rahimi et al.^14^ who have reported the adherence to appropriate radiation field size in 46.4% of cases and Tschauner et al.^15^ who showed that on average, the field size was greater than that required in 45.1% of the studied radiographs. Furthermore, Nkobley et al.^16^ indicated the field size collimation performed in 79% of the investigated children's chest radiographs, and Tohid Nia et al.^3^ reported the proper collimation of field size in the 100% of reviewed cases. According to the study of Rahimi et al.^14^, the insufficient restriction of the field size to the imaging organ, in most radiologic examinations, can be possible due to the following reasons: radiographers are not familiar with the anatomy of the exposed organ, and there is discrepancy between the light and the radiation field, and short working experience as radiographers.

An interesting point in our findings was that the radiation field sizes were appropriate to the target organs when setting the exposure factors manually, which may be due to the ability to correctly adjust the parameters according to the patients’ body size and apply the required changes. Moreover, automatic exposure control, by default, considers a size that cannot be generalised to all cases.

Furthermore, according to our results, there was no significant association between the age of the patients and the suitability of the adjusted field sizes in performing radiographies (*P*‐value> 0.05).

Considering that the limitation of the radiation field (beam collimation) to the target tissue for imaging can significantly reduce the amount of unnecessary exposure, radiation doses in the skin and internal tissues decrease.^6^, ^7^, ^8^, ^9^, ^17^ Hence, it is significantly important to observe this protection principle. The limitation of the radiation field, specifically in children's imaging, is critical as the surrounding organs are closely situated to each other. Therefore, insufficient limitation of the radiation field will increase the probability of exposure to radiation‐sensitive organs.^2^ Another impact of decreasing the size of the X‐ray field with regard to the target area in imaging is that the image receptor receives a smaller amount of scattered radiation, resulting in better contrast and quality of the final image.^2^, ^6^, ^17^

Furthermore, our results showed that the suitability of adjusted field size had a significant association with radiography type (*P*‐value = 0.03) where most appropriate field size limitation was performed for the limbs, while field size restriction was poor for the abdominal and spine imaging. Hence, the largest field sizes were more associated with the abdomen (60 × 60 cm^2^) compared to the standard values (35 × 43 cm^2^) according to Merrill's Atlas *of Radiographic Positioning & Procedures*.^12^ When the radiographers were asked about the reason of not restricting the field sizes, they were mainly concerned of missing the desired anatomy in the final radiographies and need to repeat the radiologic examinations. However, as mentioned earlier, restricting the field size reduces the volume of tissue irradiated and therefore reduces patient exposure and improves the image quality due to scatter reduction.^18^

Based on our results, protective shields were not used for patients in any of the reviewed radiographic examinations, and the lowest level of protection measures was related to the use of protective equipment for patients (0%) when various protective equipment was available in the investigated imaging centres. Our results are consistent with the literature.^3^, ^14^, ^19^ Therefore, Touhidi Nia et al.^3^ showed that the lowest level of protective measure observance by radiographers included not using of genital protective equipment, which leads to unnecessary radiation exposure to other tissues adjacent to the imaging organ. This finding was consistent with the results of the study conducted by Bezanjani,^19^ who reported a rate less than 1% of using the protective equipment for patients during radiographic examinations, and those of Rahimi et al.^14^, who indicated not using the protective equipment in most cases. Tamjidi et al.^20^ also reported the use of protective equipment at 16%.

Due to the risks of ionising radiation and the International Commission on Radiological Protection statements^11^ emphasising the use of lead shields in radiation‐sensitive organs, the use of protective equipment should be considered by authorities and healthcare professionals.^21^, ^22^, ^23^ Despite this, based on the American Association of Physicists in Medicine position statement^24^ regarding gonadal and foetal shielding, the use of protective equipment within the imaging area can conceal the anatomy or pathology of the exposed organs or lead to artefacts that may necessitate repeating the imaging. In such a situation, if the imaging process is not repeated, the interpreting physician may lose important diagnostic information. If it is repeated, it can be time consuming. Additionally, the use of protective equipment has a negative impact on automatic exposure control and image quality. All modern X‐ray systems use automatic exposure control. Therefore, the use of protective equipment in the imaging area results in increased X‐ray output, increased patient dose and impaired image quality.

The results of this study revealed that in the 26% of the reviewed radiographic examinations, the patients had at least one companion. The presence of these companions was considered necessary, because the higher attendance of the companions in the examination room was observed for patients with lower ages (*P*‐value = 0.02), specifically patients less than 20 years old. However, using the proper protective equipment (lead apron) was only performed for approximately 4% of the patient companions, and the protective measures for them were insufficient.

According to the study conducted by Tohid Nia et al.^3^, the principle of non‐closeness or non‐presence of personnel and companions during radiation was observed in 97.4% of cases. Hence, they used mechanical fixing devices to keep the patients or radiology cassettes instead of using nurses or patient companions. They also reported that the use of protective equipment for nurses and patient companions was approximately 28.9% in their cases (when their presence was significantly required). Moreover, the results of Rahimi et al.'s study^14^ indicated that the observance rate of using lead shields for patients’ companions in the radiographic examination room was 65.8%.

Based on the results, the observance rate for the various principles of radiation protection in the studied radiology departments, including limiting the field size with respect to the imaging organ, using protective equipment for the patients and their companions, and the suitability of the adjusted exposure factors for the requested radiographic examinations, had an average rate of 44.6%, which did not represent a favourable situation. Moreover, according to Rahimi et al.,^14^ the observance rate in using protective principles was approximately 71%, which is not consistent with the result of the present study. This can be due to the different levels of awareness and attention of radiographers to the hazards and harmful effects of radiation. However, according to the results of Armpilia et al.,^25^ selecting the suitable exposure factors and the radiation field collimation are important to improve the radiation protection. Radiographers who fail to adhere to the principles of radiation protection can cause unnecessary exposure to the other organs, specifically the sensitive organs, thereby increasing the probability of possible radiation side effects in the community.^26^

## Conclusion

Based on the findings, it can be concluded the observance of radiation protection principles in the studied educational hospitals was insufficient, specifically in adjusting the proper field size and the use of protective equipment for patients and their companions, which necessitates significant effort and attention. The reason may be that the educational hospitals are so crowded and radiographers have insufficient time to observe patient protection. In some cases, the radiographers do not believe in the importance of implementing the protective equipment. Therefore, continuous monitoring of radiographers’ protection performance and emphasis on the strict implementation of the protection guidelines based on the latest available standards by the relevant authorities are required. Radiographers should strictly impose the application of protective equipment, specifically for children and patient companions, and should use the mechanical devices for patients rather than patient's companion or nurses.

Furthermore, creation of educational posters and inclusion of an incentive score in the annual assessment of radiographers who strictly follow the principles of radiation protection may also be helpful. Moreover, continuous training to enhance employee knowledge, monitoring and evaluation by the authorities, and significant emphasis on qualifying university education may be necessary for improving the existing protection status and to protect the patients and their companions.

## Supporting information

Additional Supporting Information may be found in the online version of this article:

**Data S1.** Data collection sheet.
