# Supplementary figures and images for: Evaluating the principles of radiation protection in diagnostic radiologic examinations: collimation, exposure factors and use of protective equipment for the patients and their companions
Source: J Med Radiat Sci. 2020 Mar 9;67(2):119–27. doi: 10.1002/jmrs.384 (PMC7276186; doi:10.1002/jmrs.384)

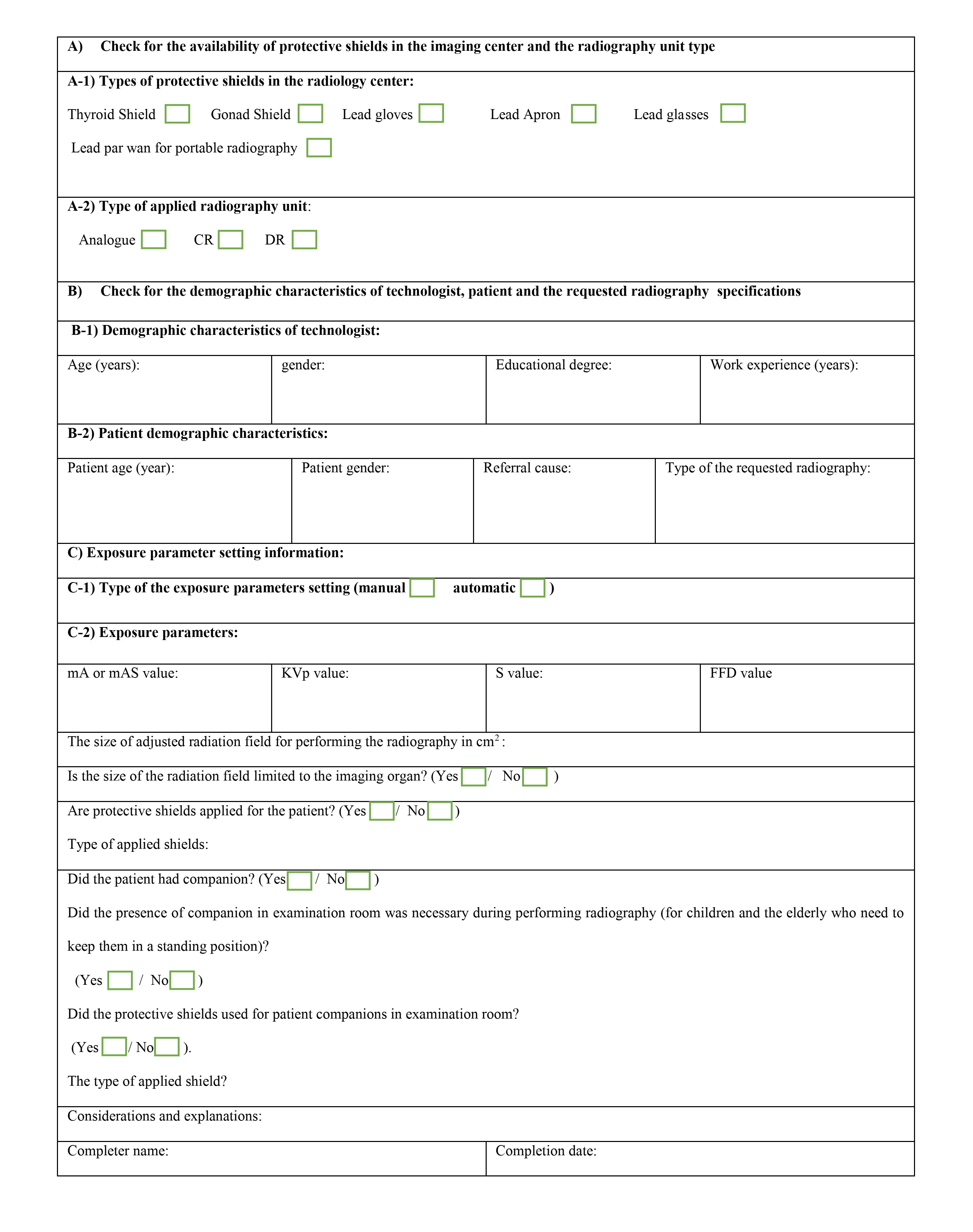

Supplement: Supplementary file 1 — Data S1. Data collection sheet. [file JMRS-67-119-s001.jpg]
